# Distinct, common and synergistic effects of insulin and IGF-1 receptors on healthy murine ageing

**DOI:** 10.1016/j.heliyon.2024.e36457

**Published:** 2024-08-17

**Authors:** Andrew MN Walker, Nicole T. Watt, Nadira Y. Yuldasheva, Sanjush Dalmia, Marcella Conning-Rowland, Chew W. Cheng, Nele Warmke, Katherine Bridge, Oliver I. Brown, Cheukyau Luk, Michael Drozd, Natalie J. Haywood, Anna Skromna, Natasha Makava, Stephen B. Wheatcroft, Mark T. Kearney, Richard M. Cubbon

**Affiliations:** Leeds Institute of Cardiovascular and Metabolic Medicine, LIGHT Laboratories, The University of Leeds, Clarendon Way, Leeds, LS2 9JT, United Kingdom

**Keywords:** Insulin, IGF-1, Healthspan, Ageing, Metabolism

## Abstract

**Objective:**

Reduced IGF-1 signalling is an evolutionarily conserved mediator of longevity, yet the magnitude of this effect is substantially larger in organisms retaining a common insulin and IGF-1 receptor. Whether this reflects the failure to simultaneously reduce IGF-1 and insulin signalling in mammalian model systems remains unexplored, as is the associated impact on markers of healthy ageing. We set out to address these uncertainties.

**Methods:**

We compared the duration of healthy life (healthspan) in male mice with haploinsufficiency of the insulin receptor (IRKO), IGF-1 receptor (IGF-1RKO), or both (DKO), versus wildtype (WT) littermates. Cognitive performance was defined using nesting studies at 3- and 24-months of age. Brain transcriptome was characterised at 3- and 18-months of age using RNA-seq.

**Results:**

Healthspan was longer in DKO versus WT, with IRKO and IGF-1RKO being intermediate. At 2 years of age, DKO also exhibited preserved nesting behaviour in contrast with all other genotypes. Differential insulin sensitivity or weight gain during ageing did not explain the preserved healthspan of DKO, since these were comparable to IRKO littermates. Brain transcriptomics at 18 months of age revealed lower expression of canonical ageing-associated genes in DKO versus WT, although many of these findings were replicated in IRKO versus WT or IGF-1RKO vs WT.

**Conclusions:**

Reduced insulin and IGF-1 receptor expression have both common and synergistic effects upon elements of healthy mammalian ageing, suggesting future ageing studies should consider targeting both insulin and IGF-1 signalling.

## Highlights

• The impact of reduced insulin and/or IGF-1 signalling on ageing were studied.
• Weight gain was predominantly mitigated by reduced insulin signalling.
• Healthspan was extended only with reduced insulin *and* IGF-1 signalling.
• Insulin and IGF-1 signalling had shared and distinct impacts on brain transcriptome.
• These mitigated canonical brain aging transcriptional changes.

## Abbreviations

DEG: Differentially Expressed Gene
DKO: Double Knockout
ELISA: Enzyme Linked Immuno-Sorbent Assay
FDR: False Discovery Rate
GO: Gene Ontology
IGF-1: Insulin-like Growth Factor-1
IGF-1R: Insulin-like Growth Factor-1 Receptor
IGF-1RKO: Insulin-like Growth Factor-1 Receptor Knockout
IR: Insulin Receptor
IRKO: Insulin Receptor Knockout
SEM: Standard Error of the Mean
WT: Wild Type

## 1 Introduction

The association between reduced insulin/IGF-1 signalling and longevity has been established in diverse model organisms using genetic, pharmacological and dietary interventions [1,2], prompting interest in this as a paradigm to extend human lifespan. However, the striking observations made in genetically modified invertebrates, which share a common insulin and IGF-1 receptor, have been subtler in mammalian model systems with isolated targeting of insulin or IGF-1 receptors [3]. For example, global IGF-1 receptor haploinsufficiency has been shown to extend lifespan by 33 % in female mice, with a non-significant 16 % extension in male mice [4]. Similarly, male and female mice deficient for the growth hormone receptor (leading to reduced IGF-1 expression) also experience an approximate 30 % lifespan extension [5]. Less marked lifespan extension has been noted in male mice lacking one insulin receptor allele [6], and mice of both sexes null for insulin receptors in adipose tissue [7]. Knockout of shared signalling nodes downstream of insulin and IGF-1 receptors (such as IRS1, S6K1 and FoxO 3) has also been associated with similarly modest lifespan extension [[8], [9], [10]], along with reduced cognitive decline and neuroinflammation during aging [11,12].

Whether the discrepancies between vertebrate and invertebrate models reflect a failure to simultaneously target the functionally overlapping insulin and IGF-1 signalling apparatus remains unknown and is an important barrier to developing effective strategies to promote healthy ageing. Moreover, it is increasingly appreciated that extension of lifespan may come at the expense of extending time with poor health, resulting in a focus on interventions that prolong healthy life, or healthspan [13]. The literature describing whether reduced insulin and IGF-1 signalling protects against ageing-associated functional decline is sparse, particularly when applied to genetic interventions in mammalian models. Hence, we set out to study whether reduced insulin and/or IGF-1 receptor expression extend healthy life in mice.

## 2 Materials and methods

### 2.1 Acquisition, breeding and husbandry of mice

Mice were bred onto a C57BL/6J background for >10 generations. Cages were maintained in humidity and temperature-controlled conditions (humidity 55 % at 22 °C) with a 12-h light–dark cycle. A standard chow diet (Beekay BK001E, B&K Universal Limited) was provided, which contained 4.7 % fat, 18.7 % protein and 59.7 % nitrogen free extract (16.3 kJ/g). As previously described [14], male insulin receptor haploinsufficient mice (IRKO) were crossed with female IGF-1 receptor haploinsufficient mice (IGF-1RKO), resulting in progeny with the following genotypes: 1) Wild-type (WT); 2) insulin receptor haploinsufficient (IRKO); 3) IGF-1 receptor haploinsufficient; and 4) insulin and IGF-1 receptor haploinsufficient (DKO). IRKO mice were generated as described by Accili et al., by introducing a premature stop mutation in exon 4 of the Insr gene [15]. IGF-1RKO were generated by Holzenberger et al., using homologous recombination to deleted exon 3 of the Igf1r gene [4]. 15 male mice per genotype were observed during assessment of healthspan. In a separate cohort, male mice were killed at 3 or 18-months old to collect brain tissue for transcriptomic analysis. All procedures were performed according to accepted standards of humane animal care, approved by the ethical review committee of the University of Leeds, and conducted under license from the United Kingdom Home Office.

### 2.2 Metabolic assessment

Mice were fasted overnight prior to glucose tolerance testing or for 2 h prior to insulin tolerance testing. Whole capillary blood was sampled from tail vein, with glucose concentrations determined in whole blood by a portable meter (Roche Diagnostics, UK). Glucose and insulin tolerance tests were performed by blood sampling after an intraperitoneal injection of glucose (1 mg/g; Sigma-Aldrich, UK) or human recombinant insulin (0.75 units/kg, Actrapid; Novo Nordisk, Denmark), respectively [14].

### 2.3 Healthspan endpoints

Assessment of healthspan was made according to criteria provided by a Home Office approved Veterinary Surgeon, based upon published literature [16], to ensure animal welfare. Animals were considered to have reached their healthspan endpoint if one or more of the following conditions was met: 1) Spontaneous death before one of the following endpoints; 2) Body condition score ≤2 out of 5 [16]; 3) Body weight loss of ≥15 % of the average highest body weight, sustained for at least two consecutive weeks; 4) Hunched posture/starry coat/abnormal gait of more than 48 h duration; 5) Any progressively enlarging subcutaneous lump/swelling; 6) Excessive hair loss, monitored over at least one week. Assessment to confirm whether an animal had met a healthspan endpoint was made by two independent observers except in the case of spontaneous death or body weight loss of ≥15 % of the average highest body weight; all assessments were made blind to genotype. Animals were culled in accordance with Schedule 1 of The Animals (Scientific Procedures) Act 1986 (Amended 2012) once a healthspan endpoint was reached. In keeping with our United Kingdom Home Office Project License (P144DD0D6) stipulations, any animals considered to be experiencing excessive pain or distress (outside of the criteria mentioned above) were culled after assessment by two independent observers blinded to genotype.

### 2.4 Nesting studies

Mice were caged individually and left overnight with a nestlet. The next morning the cage was examined for the presence of a nest and images taken to quantify nest building, according to an established validated protocol [17]. Nest photographs were taken by a blinded researcher, and subsequently scored by 4 genotype-blinded researchers per mouse, to derive a mean nesting score for each mouse. Scoring criteria were as follows: 1) Nestlet not noticeably touched (more than 90 % intact); 2) Nestlet partially torn (50–90 % remaining intact); 3) Nestlet mostly shredded but often no identifiable nest site: less than 50 % of the Nestlet remains intact, but less than 90 % is within a quarter of the cage floor area; i.e., the cotton is not gathered into a nest but is spread around the cage. The material may sometimes be in a broadly defined nest area, but the critical definition here is that 50–90 % has been shredded; 4) An identifiable but flat nest: more than 90 % of the Nestlet is torn and the material is gathered into a nest within a quarter of the cage floor area, but the nest is flat, with walls higher than mouse body height (of a mouse curled up on its side) for less than 50 % of its circumference; 5) A (near) perfect nest: more than 90 % of the Nestlet is torn and the nest is a crater, with walls higher than mouse body height for more than 50 % of its circumference.

### 2.5 Immunoblotting

Mice aged 3 or 18 months old underwent terminal isoflurane anesthesia followed by exsanguination via the inferior vena cava. The gastrocnemius muscles and brain (including cerebellum and brain stem) were harvested and hemisected longitudinally; the left side was used for these experiments. Tissues were mechanically pulverized (Qiagen TissueLyser II) in lysis buffer (Thermo, Extraction buffer, FNN0011) and protein content was quantified by a BCA assay (Sigma-Aldrich, St. Louis, MO). One-hundred micrograms of protein were resolved on a 4–12 % Bis-Tris gel (Bio-Rad, Hertfordshire, UK) and transferred to nitrocellulose membranes. Membranes were probed with antibodies diluted in 5 % BSA (Insulin receptor - Cell Signaling Technology #3025S [1:100 dilution]; IGF-1 receptor - Cell Signaling Technology #9750 [1:100 dilution]; Serine-473 phospho-Akt - Cell Signaling Technology #4060S [1:1000 dilution]; total Akt - Cell Signaling Technology #9272S [1:1000 dilution]; beta-actin - Santa Cruz Biotech sc-47778 [1:3000 dilution]), before incubation with appropriate secondary horseradish peroxidase-conjugated antibody. Blots were visualised with Immobilon Western Chemiluminescence HRP Substrate (Merck Millipore, Hertfordshire, UK) and imaged with Syngene chemiluminescence imaging system (SynGene, Cambridge, UK). Densitometry was performed in ImageJ (NIH, Bethesda).

### 2.6 Serum insulin and IGF-1

Non-fasting serum was prepared from venous blood and stored at −80 °C until use. Enzyme linked immuno-sorbent assays (ELISA) were performed according to manufacturers’ instructions to measure insulin (CrystalChem Ultrasensitive mouse insulin ELISA - code 90080) and IGF-1 (R&D systems - code MG100).

### 2.7 Brain transcriptomics

Mice aged 3 or 18 months old underwent terminal isoflurane anesthesia followed by exsanguination via the inferior vena cava. The brain (including cerebellum and brain stem) was harvested and hemisected longitudinally; the right side was mechanically pulverized (Qiagen TissueLyser II) before using the New England Biolabs Monarch Total RNA Miniprep Kit to isolate RNA and perform DNAse I treatment. RNA-seq was performed by the University of Leeds Next Generation Sequencing core facility (Illumina NextSeq 2000) to acquire 150 base pair single-end reads. Raw data have been deposited at ArrayExpress (https://www.ebi.ac.uk/biostudies/arrayexpress) under accession ID E-MTAB-13481. Quality control of the raw sequences were performed using FastQC v.0.11.4 to evaluate the overall quality [18]. Adapter sequences were trimmed from raw reads (TrimGalore v.0.6.6) [19], before alignment to the mouse genome (GRCm39) using STAR aligner v.2.7.10a followed by featureCounts (Subread v2.0.1) to derive gene count data [20]. Subsequent bioinformatics were conducted using DESeq2 v.1.36.0 in the R environment (v4.2.0) for differential gene expression analysis [21], and g:Profiler (https://biit.cs.ut.ee/gprofiler/gost) for defining enriched Gene Ontology (GO) terms amongst differentially expressed genes (DEGs). Multiple testing was accounted for using Benjamini-Hochberg false discovery rate (FDR) adjusted p values, both when defining DEGs and enriched GO terms. Heatmap illustration used Pheatmap [22] with row scaling, and Venn diagrams were designed using InteractiVenn [23].

### 2.8 Statistics

Data are presented as mean ± SEM. All genotypes were compared with ANOVA (and post hoc Tukey) or Kruskal-Wallis (and post hoc Dunn) tests, as appropriate. Specified two genotype comparisons were made using t-tests or Mann-Whitney U tests, as appropriate. Healthspan was illustrated with Kaplan-Meier curves and compared with log-rank resting. Statistical significance was defined as p < 0.05.

## 3 Results

**Fig. 1:**
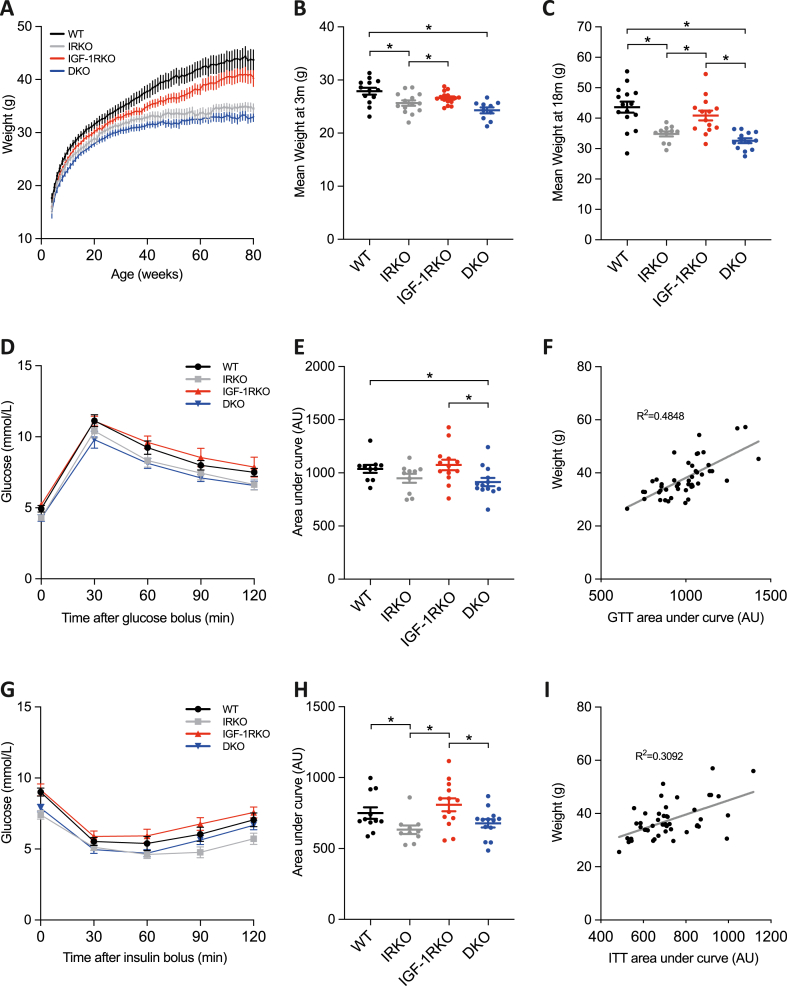
Metabolic characterisation during ageing A) Body mass during ageing (n = 15/genotype); B) Body mass at 3 months (ANOVA p < 0.001; n = 15,14,15,14); C) Body mass at 18 months (ANOVA p < 0.001; n = 15,11,14,13); D,E) Glucose tolerance testing at 20 months, quantified by area under curve (ANOVA p = 0.03; n = 10,10,13,13); F) Correlation between area under glucose tolerance test curve and body mass (p < 0.001; n = 46); G,H) Insulin tolerance testing at 20 months, quantified by area under curve (ANOVA p = 0.01; n = 11,10,13,13); I) Correlation between area under insulin tolerance test curve and body mass (p < 0.001; n = 47). In panels A–E and G-H data are presented as mean (SEM). * denotes p < 0.05. AU denotes arbitrary units.

As previously described [14], we bred insulin receptor haploinsufficient mice with IGF-1 receptor haploinsufficient mice, producing progeny with the following genotypes: wild-type (WT); insulin receptor haploinsufficient (IRKO); IGF-1 receptor haploinsufficient (IGF-1RKO); insulin and IGF-1 receptor haploinsufficient (DKO). Male littermates (n = 15/genotype) were then fed a standard chow diet and observed by researchers blinded to genotype until spontaneous death or an a priori defined humane endpoint described earlier. All genotypes gained weight during adulthood (Fig. 1A), with mean weight being significantly less in IRKO and DKO than WT, both at 3- and 18-months of age (Fig. 1B and C). At 20 months of age, this was associated with increased glucose tolerance (Fig. 1D and E) and increased insulin sensitivity (Fig. 1G and H) in all surviving IRKO and DKO versus WT and IGF-1RKO littermates. Notably, body mass across genotypes correlated with glucose tolerance (R^2^ = 0.48; Fig. 1F) and insulin sensitivity (R^2^ = 0.31; Fig. 1I). Glucose- and insulin-tolerance in young mice were not assesses as these have previously been published [14].

**Fig. 2:**
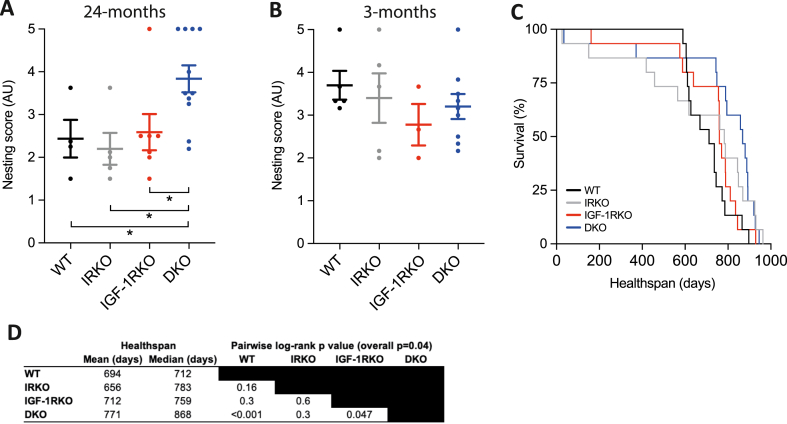
Healthspan is extended in DKO mice A) Mean nesting score at 24 months (Kruskal-Wallis p = 0.01; n = 4,5,7,11); B) Mean nesting score at 3 months (Kruskal-Wallis p = 0.42; n = 5,3,3,9); C) Kaplan-Meier curve illustrating healthspan (Log rank p = 0.04; n = 15/genotype). D) Summary data from Kaplan-Meier curves and pairwise log-rank tests between specified genotypes. In panels A–B data are presented as mean (SEM). * denotes p < 0.05. AU denotes arbitrary units.

Nesting studies were then performed in all mice surviving to 24 months of age, as a marker of behaviour and global cognitive performance. The mean nesting quality score allocated by 4 blinded assessors using a validated methodology [17] was significantly different between genotypes, with DKO exhibiting clearly superior performance against other groups (Fig. 2A). Notably, nesting performance did not correlate with body mass, and nesting scores in a subgroup of 3-month old mice from this colony demonstrated that all genotypes produced high quality nests (Fig. 2B). Importantly, the superior nesting scores of DKO were also associated with extended survival free from markers of ill health that mandated euthanasia according to our humane endpoint protocol (Log rank p = 0.04 across all genotypes; Fig. 2C). When comparing individual genotypes, only DKO survived significantly longer than WT (Log rank p < 0.001; median survival 868 versus 712 days), with survival of IRKO and IGF-1RKO groups being intermediate (Fig. 2D).

**Fig. 3:**
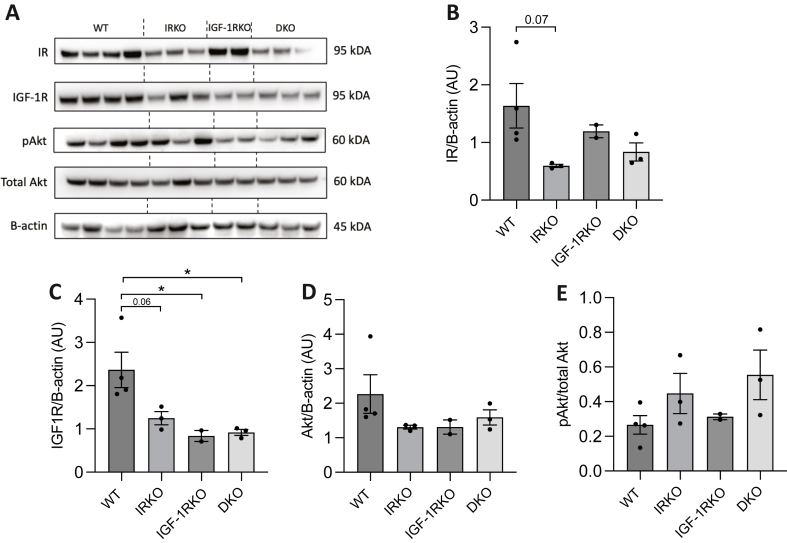
Immunoblotting of whole brain from 3-month old WT, IRKO, IGF-1RKO and DKO A) Immunoblots of insulin receptor (IR), IGF-1 receptor (IGF-1R), Serine-473 phosphorylated Akt (pAkt), total Akt and beta-actin from wild type (WT), insulin receptor knockout (IRKO) IGF-1R knockout (IGF-1RKO) and double knockout (DKO) mouse brain at 3-months of age (n = 3,3,3,2). B) Densitometry quantification of IR expression normalised to beta-actin; C) Densitometry quantification of IGF-1R expression normalised to beta-actin; D) Densitometry quantification of total Akt expression normalised to beta-actin; E) Densitometry quantification of pAkt expression normalised to total Akt. In panels B–E data are presented as mean (SEM); * denotes p < 0.05 (Student's t-test), whilst p values approaching statistical significance are specifically quantified above the relevant comparator bars; AU denotes arbitrary units.

**Fig. 4:**
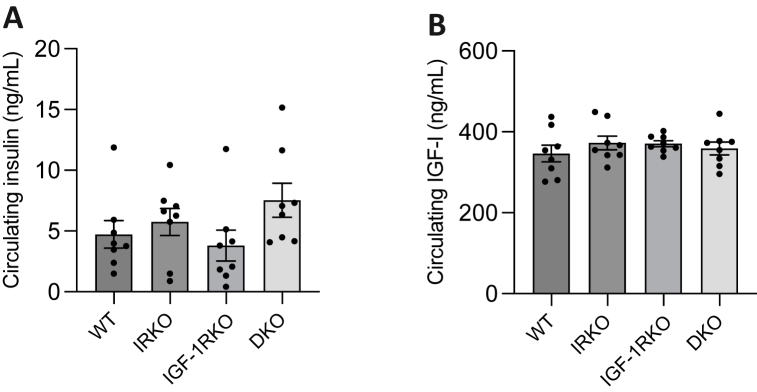
Serum insulin and IGF-1 in 18-month old WT, IRKO, IGF-1RKO and DKO Serum non-fasting insulin (A) and IGF-1 (B) concentrations defined by ELISA in wild type (WT), insulin receptor knockout (IRKO) IGF-1R knockout (IGF-1RKO) and double knockout (DKO) mice at 18-months of age. Data are presented as mean (SEM). There are no statistically significant between-group differences (Mann-Whitney test).

To explore the molecular correlates of preserved nesting performance (which may imply preserved cognitive function) in aged DKO versus aged WT, we collected brain tissue from a separate batch of mice. Mice at 3-months were included to define differences in youth, whilst mice at 18-months were used to define differences in older age before mortality rates diverged between genotypes. First, we explored protein expression of the insulin receptor (IR) and IGF-1 receptor (IGF-1R) in the brain of 3-month (Supplemental Fig. 1) and 18-month (Fig. 3) old mice from all genotypes using immunoblotting. This revealed expected patterns of receptor expression, with IR being lower in IRKO and DKO than WT and IGF-1RKO, and IGF-1R being lower in IGF-1RKO and DKO versus WT and IRKO. Downstream signalling, inferred by the ratio of Serine-473 phosphorylated Akt to total Akt (pAkt/Akt), was increased in all genotypes versus WT (with IGF-1RKO versus WT p = 0.055) at 3-months. At 18-months, pAkt/Akt was nominally greater than WT in only IRKO and DKO, although neither reached statistical significance in this small sample (n = 2–4 per genotype-age group). We also examined skeletal muscle from 18-month old mice, again finding expected patterns of receptor expression in each genotype, although without the higher pAkt/Akt seen in brain of DKO and IRKO (Supplemental Fig. 2). We also defined the non-fasting circulating insulin and IGF-1 concentrations of all genotypes at 18-months (Fig. 4). This revealed non-significantly greater serum insulin concentrations in IRKO and DKO than WT and IGF-1RKO, whilst serum IGF-1 was similar in all genotypes.

**Fig. 5:**
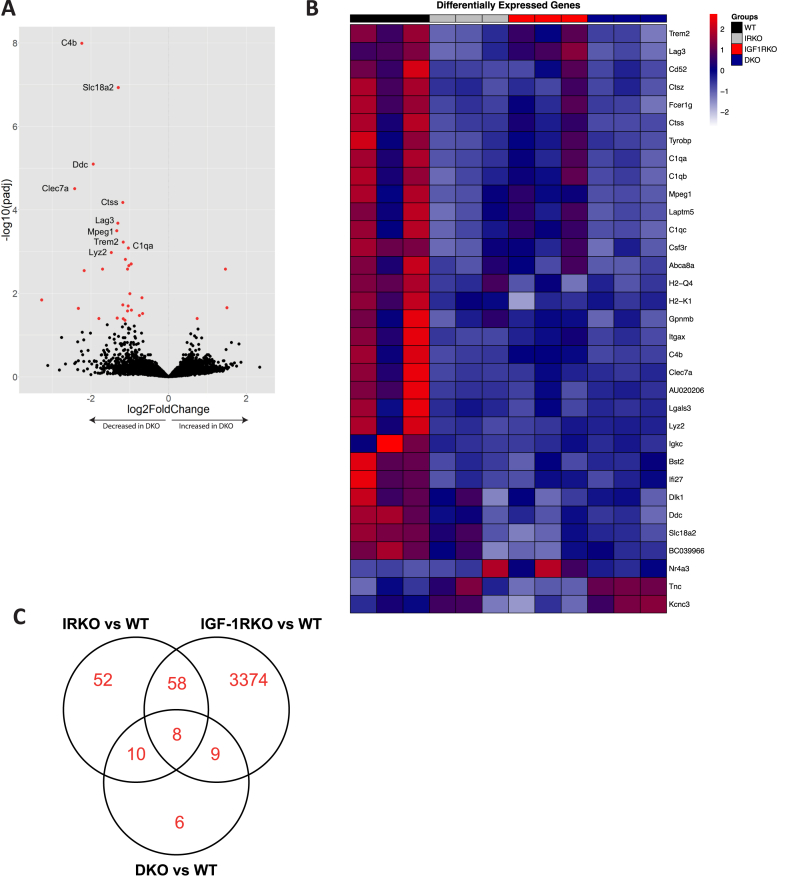
Brain transcriptomic characterisation A) Volcano plot illustrating differentially expressed genes (DEGs) in 18-month old DKO versus 18-month old WT mice. Log_2_ fold change data below zero denote lower expression in DKO than WT. Red data points denote 33 DEGs with FDR-adjusted p value < 0.05, with the top 10 labelled by gene name. B) Heatmap of 33 DEGs between 18-month old DKO versus WT, with IRKO and IGF-1RKO data presented to illustrate shared and distinct patterns using hierarchical clustering of rows. Data are normalised by gene with expression measured in arbitrary units. Unadjusted data are presented in Table 1. C) Venn diagram illustrating common and unique DEGs from comparisons of DKO vs WT, IRKO vs WT and IGF-1RKO vs WT.

To extend the molecular phenotyping of the brain, we also performed RNA-sequencing in 3 mice per genotype, both at 3-months and 18-months of age. We first defined insulin receptor (Insr) and IGF-1 receptor (Igf1r) expression and observed these to be higher in IRKO, IGF-1RKO and DKO versus WT (Supplemental Table 1). This is likely to reflect compensatory overexpression, including of mutant alleles in which only Insr exon 4 and/or Igf1r exon 3 are deleted; importantly this is not reflected at protein level where appropriate deletion was noted (Fig. 3). The small size of Insr exon 4 and Igf1r exon 3 means that a negligible number of reads map to these even in non-mutant mice and so we cannot evaluate differential expression of these exons between genotypes. We then focussed on aged DKO versus WT and found 33 DEGs (30 down- and 3 up-regulated) at FDR p < 0.05 (Fig. 5A, Table 1 and Supplemental Table 2). Enriched GO terms amongst these 33 DEGs (Supplemental Table 3) included inflammatory and neuronal processes such as ‘Leukocyte mediated immunity’ (FDR p = 9.8 × 10^−15^), ‘Complement component C1 complex’ (FDR p = 1.4 × 10^−7^) and ‘Aminergic neurotransmitter loading into synaptic vesicle’ (FDR p = 2.0 × 10^−4^). We then asked whether these 33 genes had been identified in a published transcriptomic meta-analysis of mammalian brain ageing [24]. None of the 16 downregulated genes in this meta-analysis (Supplemental Table 4) corresponded with DEGs in aged DKO versus WT. However, 12 of the 33 (36.4 %, Chi-squared p < 0.001; listed in Table 1) were present in the 147 significantly upregulated genes in this meta-analysis (Supplemental Table 4); all were lower in DKO than WT. This suggests that canonical transcriptomic features of brain ageing are diminished in DKO.

**Table 1:** Differentially expressed genes in brain of 18-month old WT versus 18-month old DKO mice.

Gene	WT	IRKO	IGF-1RKO	DKO	Ageing meta-analysis
Abca8a	21.9 (2.9)	12.8 (2.1)	14.9 (2.5)	10.2 (0.5)*	
Bst2	14.4 (2.2)	4.7 (0.5)*	4.8 (1.5) *	6.4 (0.8)*	
C1qa	92.2 (11.3)	46.2 (2.5)*	66.8 (8.3)	45.4 (3.1)*	Up
C1qb	78.5 (9.0)	42.6 (4.0)*	62.1 (6.2)	40.6 (2.6)*	Up
C1qc	67.4 (9.5)	36.8 (3.8)	54.0 (2.5)	33.4 (1.4)*	Up
C4b	97.9 (24.5)	21.9 (2.0)*	45.9 (2.5)*	20.9 (1.7)*	
Cd52	6.2 (1.1)	1.7 (0.1)*	3.3 (1.1)	1.9 (0.3)*	
Clec7a	7.2 (1.8)	0.8 (0.1)*	1.8 (0.4)*	1.3 (0.1)*	Up
Csf3r	6.9 (0.3)	3.4 (0.7)	4.7 (0.5)	3.2 (0.4)*	
Ctss	125.6 (18.4)	59.1 (3.0)*	86.1 (7.9)	55.8 (0.5)*	Up
Ctsz	74.3 (4.7)	47.2 (1.7)*	53.1 (5.8)	46.5 (2.2)*	Up
Ddc	35.7 (2.9)	13.8 (4.0)	12.1 (2.8)*	9.4 (2.4)*	
Dlk1	76.0 (10.9)	39.4 (15.2)	30.9 (8.5)*	34.1 (7.1)*	
Fcer1g	17.1 (1.4)	9.0 (0.3)*	12.3 (1.6)	8.3 (0.7)*	Up
Gpnmb	6.6 (1.6)	3.0 (1.0)	3.2 (0.2)*	1.9 (0.6)*	Up
H2-K1	69.8 (5.9)	47.2 (2.4)	37.1 (5.8)*	44.2 (1.5)*	
H2-Q4	9.7 (0.5)	6.1 (1.0)	4.8 (0.9)*	5.0 (0.4)*	
Ifi27	71.8 (9.9)	31.0 (2.9)*	36.7 (4.4)*	36.2 (3.7)*	
Igkc	3.5 (1.2)	0.5 (0.1)*	0.7 (0.2)*	0.7 (0.1)*	
Itgax	1.8 (0.5)	0.2 (0.05)*	0.6 (0.1)	0.2 (0.1)*	
Kcnc3	358 (39)	385 (130)	164 (63)*	599 (49)*	
Lag3	10.4 (0.7)	4.6 (0.7)*	10.2 (0.9)	4.2 (0.4)*	
Laptm5	46.0 (5.0)	29.6 (3.4)	38.2 (2.8)	27.5 (0.5)*	Up
Lgals3	10.8 (3.7)	2.8 (0.4)*	2.0 (1.2)*	2.4 (0.2)*	Up
Lyz2	39.7 (9.1)	10.4 (1.4)*	12.1 (2.0)*	14.4 (0.4)*	
Mpeg1	40.9 (7.3)	19.9 (3.9)	28.4 (2.0)	16.3 (0.5)*	
BC039966	5.1 (0.4)	2.1 (1.1)	1.2 (0.6)*	2.1 (0.3)*	
AU020206	10.7 (1.5)	4.8 (0.1)*	4.8 (0.7)*	5.2 (0.2)*	
Nr4a3	8.6 (1.8)	36.4 (24.7)	57.4 (15.2)*	23.8 (3.6)*	
Slc18a2	42.9 (1.7)	25.9 (6.4)	14.0 (4.7)*	17.6 (1.9)*	
Tnc	5.1 (1.6)	11.0 (2.8)	2.6 (0.3)	14.7 (0.3)*	
Trem2	22.6 (1.8)	10.5 (1.1)*	18.3 (1.7)	10.1 (1.2)*	Up
Tyrobp	29.8 (4.9)	14.1 (1.1)*	21.3 (2.4)	14.6 (0.8)*	Up

List of differentially all expressed genes (DEGs) in the brain of 18-month DKO vs. WT. For each gene, data are presented from WT, IRKO, IGF-1RKO and DKO brain at 18-months (n = 3 per genotype). Data are DESeq2 derived normalised counts per million presented as mean (SEM), with * denoting Benjamini-Hochberg FDR-adjusted p value < 0.05 versus WT. The ‘Ageing meta-analysis’ column highlights genes identified as differentially expressed in old versus young brain in the ageing transcriptome meta-analysis of Palmer et al. [24], describing the direction of differential expression in old age.

Next, we explored expression of the 33 DEGs (between DKO and WT) in all aged genotypes to understand the potential contributions of diminished insulin and/or IGF-1 signalling to the differential gene expression found in DKO. This revealed broadly similar expression patterns in aged IRKO and DKO, whereas IGF-1RKO was most often intermediate to DKO and WT (Fig. 5B and Table 1). Of the 33 DEGs, Kcnc3 exhibited greatest dissimilarity between DKO and all other genotypes; but this did not reach statistical significance when comparing IRKO with DKO (p = 0.09). To more systematically define potential contributions of insulin and IGF-1 signalling to the DEGs observed in DKO versus WT, we contrasted the DEGs between IRKO versus WT, along with IGF-1RKO versus WT (Fig. 5C and Table 1). Of the 128 DEGs in IRKO versus WT (Supplemental Table 5), 18 were also DEGs in DKO versus WT; enriched GO terms amongst these 18 genes are listed in Supplemental Table 6. Of the 3449 DEGs in IGF-1RKO versus WT (Supplemental Table 7), 17 were also DEGs in DKO versus WT; enriched GO terms amongst these 18 genes are listed in Supplemental Table 8. Notably, 8 DEGs were common to DKO versus WT, IRKO versus WT, and IGF-1RKO versus WT (Table 1); enriched GO terms amongst these 18 genes are listed in Supplemental Table 9. These data suggest that there may be shared and distinct contributions of insulin and IGF-1 signalling to the DEGs and GO terms noted in DKO versus WT.

Finally, we performed analyses of young (3-month old) brain tissue. When comparing aged WT to young WT, we identified no DEGs reaching statistical significance after correcting for multiple testing (Supplemental Table 10), possibly reflecting an underpowered analysis. However, 271 genes reached unadjusted p < 0.05, and 48 of these were present the published transcriptomic meta-analysis of mammalian brain ageing noted earlier [24]. We then compared young DKO versus young WT and found 76 DEGs (Supplemental Table 11). Only 6 of these DEGs were also present in the 33 DEGs from aged DKO versus WT (C4b, Clec7a, Lyz2, Nr4a3, Lgals3, H2-K1); this may indicate that these 6 genes differed throughout life, rather than denoting altered brain ageing in DKO. Enriched GO terms in the 76 DEGs from young DKO versus WT were also different from those found in aged DKO versus WT (Supplemental Table 12). These data suggest that most transcriptional differences noted in aged DKO versus WT were not simply enduring differences already present in youth.

## 4 Discussion

Our data show for the first time that genetically reduced insulin and IGF-1 receptor expression in male mice extends healthspan, preserves cognitive performance in old age and alters brain transcriptional features associated with ageing. Isolated reduction in insulin or IGF-1 receptor expression was insufficient to significantly extend healthspan parameters in male mice, suggesting a synergistic effect, possibly reflecting functional compensation between these evolutionarily related receptors [25]. Our metabolic characterisation suggests that reduced body mass and increased insulin sensitivity, two parameters often associated with longevity [26], are not sufficient to denote healthy ageing, since the similar metabolic phenotype of IRKO and DKO was not mirrored in their healthspan. It is striking that IRKO and DKO mice exhibited evidence of metabolic insulin sensitisation, in spite of lower receptor expression, although this may be accounted for by their lower body mass, rather than increased insulin signalling. We observed another potential paradox in brain Akt phosphorylation, which is greater in IRKO and DKO than WT at 3-months (and remained non-significantly higher in the brain at 18-months, but this was not apparent in skeletal muscle at 18-months). Akt is a critical downstream signalling node of insulin and IGF-1 receptors, along with many other receptors. Diminution of Akt signalling has been linked to longevity [27], but insulin signalling is thought to be important for brain function and during health brain ageing [28]. However, it is likely that there are complex relationships between insulin/IGF-1 signalling, Akt activation, and ageing [29], which may also differ between organs, and so this issue requires ongoing research. Moreover, it is important to note that reduced insulin and/or IGF-1 signalling noted in other contexts, such as type 2 diabetes mellitus, are highly distinct from inherited reduction in insulin and/or IGF-1 receptor expression, making these difficult to compare. Notably, we also found known ageing-associated transcriptomic signatures to be diminished in aged DKO versus WT. However, the differentially expressed genes observed in aged DKO versus WT were often similarly altered in IRKO versus WT, and to a lesser extent by IGF-1RKO versus WT, implying that isolated reduction of brain insulin or IGF-1 receptor expression could drive many of these differences. Importantly, as the healthspan and cognitive performance of aged DKO was distinct from IRKO and IGF-1RKO, many of the differentially expressed genes we noted in DKO appear unlikely to explain their phenotype. An outlier in the aged brain transcriptomic data was Kcnc3, a potassium-conducting ion channel expressed abundantly in neurons, which was higher in DKO than WT, but comparable in IRKO and WT (Fig. 5C). Interestingly, the Kcnc3 gene was identified as one of 19 to be differentially methylated in multiple tissues during human ageing [30]. Moreover, Kcnc3 loss of function mutations cause learning defects in mice [31], and neurodegeneration in humans [32]. Further studies will be required to explore the potential relevance of this gene and to identify others requiring combined diminution of insulin and IGF-1 receptor expression. More broadly, our finding that reduced brain insulin and/or IGF-1 receptor expression diminishes established ageing associated transcriptional changes is an important finding for further exploration.

Whilst our preliminary study has made novel and potentially important observations, it is important to acknowledge its limitations. Studies linking reduced insulin or IGF-1 signalling to murine longevity and stress resistance have found sexual dimorphism [4,6,[33], [34], [35], [36]]. Indeed, the existing literature shows that findings must not be generalised between sexes [37]; hence it will be very important for future studies to examine female DKO mice, rather than generalising our observations from male mice. It is also important to emphasise that our healthspan data indicate that prior to 600 days of age, WT mice were less likely than DKO to exhibit poor heath, with this pattern reversing after this time, with DKO overall having longer healthspan. This may suggest a trade-off between health in youth and old age, potentially relating to a hormesis phenomenon [38], and this potential limitation requires further exploration in future studies. It will also be important for future studies of DKO mice to explore transcriptional differences in defined brain regions, or even cell lineages, given the major heterogeneity in physiology and age-related changes between regions. Indeed, whilst our whole-brain RNA-seq data highlight many hits identified in a large meta-analysis of brain ageing, suggesting our hits are likely to be valid, our approach may not have identified important regional differences; the small sample size is also likely to limit our power to detect important differences in our molecular studies, as noted when comparing RNA-seq data from young versus old WT brain. Moreover, it will be important for future studies to conduct a broader repertoire of physiological and molecular characterisation across multiple organ systems in larger samples to address the generalisability of our observations to the wider consequences of ageing. For example, phospho-proteomics may provide unbiased assessment of the important signalling downstream consequences of insulin receptor and/or IGF-1 receptor haploinsufficiency, which our current Western blotting data cannot define. This will be particularly important in comprehensively defining distinct, common and synergistic elements of insulin and IGF-1 signalling in youth and old age. It is also important to emphasise that our nesting performance data do not comprehensively or specifically assess domains of cognition and motor function. Therefore, our data must be viewed as preliminary, requiring validation with other methods like Y-maze and object recognition testing, along with measures of strength and agility [39].

## 5 Conclusions

In summary, our data suggest both common and synergistic effects of insulin and IGF-1 signalling during ageing. Future ageing research assessing the broader implications of simultaneous reduction of insulin and IGF-1 receptor expression may inform the development of more effective therapeutic approaches to extend healthy life.

## Funding

10.13039/501100000274British Heart FoundationFS/14/10/30472 and 10.13039/501100000691Academy of Medical Sciences SGL022\1028.

## Data availability

Our RNA-sequencing raw data are available from ArrayExpress under accession number E-MTAB-13481 (https://www.ebi.ac.uk/biostudies/arrayexpress).

## CRediT authorship contribution statement

**Andrew MN Walker:** Writing – original draft, Investigation, Funding acquisition, Formal analysis, Conceptualization. **Nicole T. Watt:** Writing – review & editing, Investigation, Formal analysis, Conceptualization. **Nadira Y. Yuldasheva:** Writing – review & editing, Investigation, Formal analysis. **Sanjush Dalmia:** Writing – review & editing, Investigation, Formal analysis, Conceptualization. **Marcella Conning-Rowland:** Writing – review & editing, Investigation, Formal analysis. **Chew W. Cheng:** Writing – review & editing, Investigation, Formal analysis. **Nele Warmke:** Writing – review & editing, Investigation. **Katherine Bridge:** Writing – review & editing, Investigation. **Oliver I. Brown:** Writing – review & editing, Investigation, Formal analysis. **Cheukyau Luk:** Writing – review & editing, Investigation. **Michael Drozd:** Writing – review & editing, Investigation. **Natalie J. Haywood:** Writing – review & editing, Investigation. **Anna Skromna:** Writing – review & editing, Investigation. **Natasha Makava:** Writing – review & editing, Investigation. **Stephen B. Wheatcroft:** Writing – review & editing, Resources, Project administration, Conceptualization. **Mark T. Kearney:** Writing – review & editing, Resources, Funding acquisition, Conceptualization. **Richard M. Cubbon:** Writing – original draft, Investigation, Funding acquisition, Formal analysis, Conceptualization.

## Declaration of competing interest

None.
