# Supplementary figures and images for: Distinct, common and synergistic effects of insulin and IGF-1 receptors on healthy murine ageing
Source: Heliyon. 2024 Aug 17;10(16):e36457. doi: 10.1016/j.heliyon.2024.e36457 (PMC11379992; doi:10.1016/j.heliyon.2024.e36457)

**A**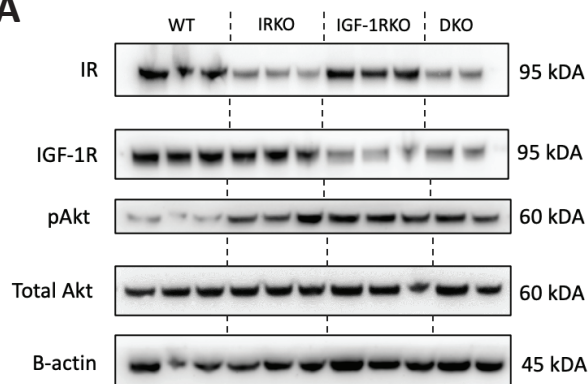**B**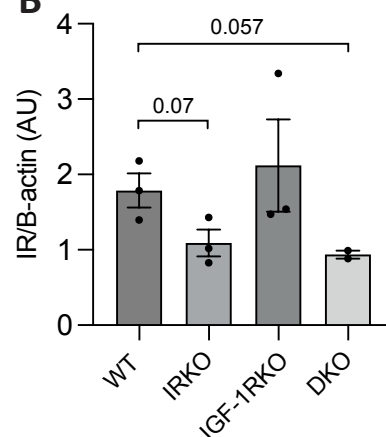**C**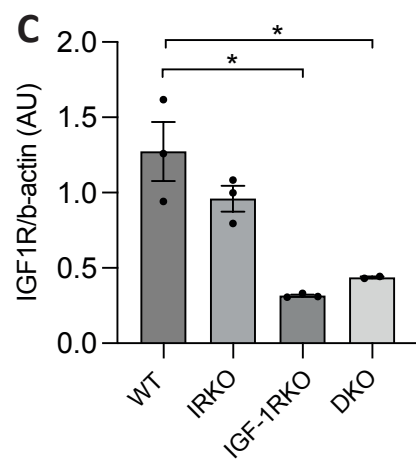**D**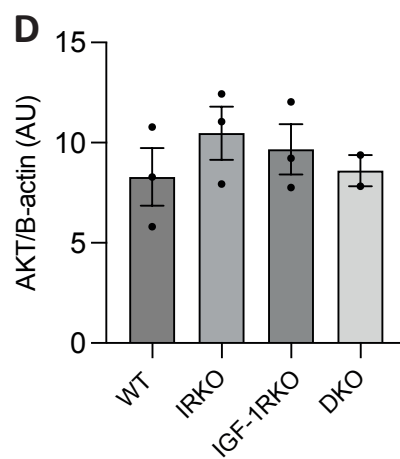**E**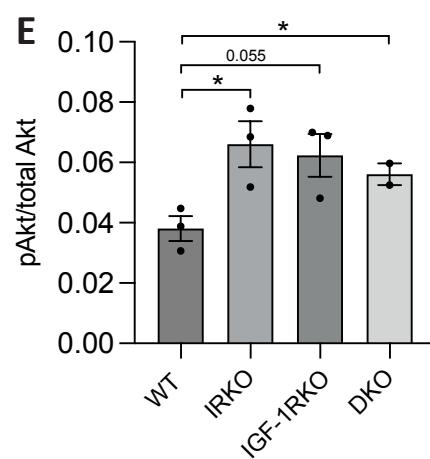

**A**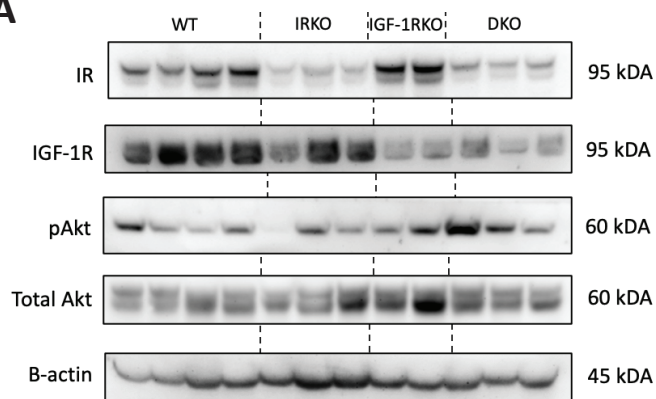**B**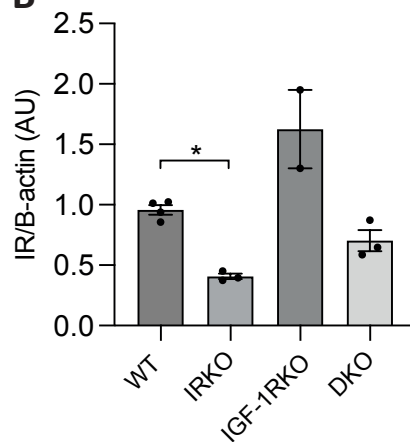**C**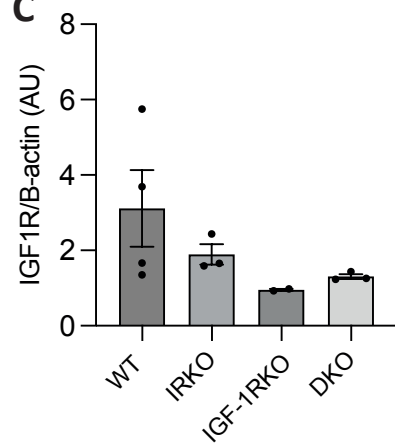**D**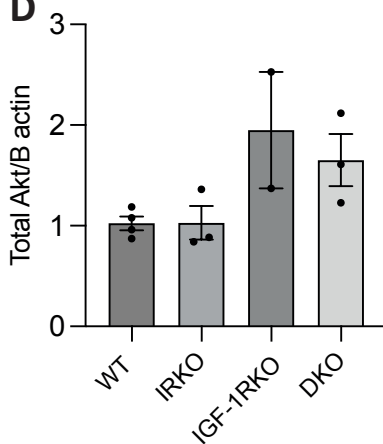**E**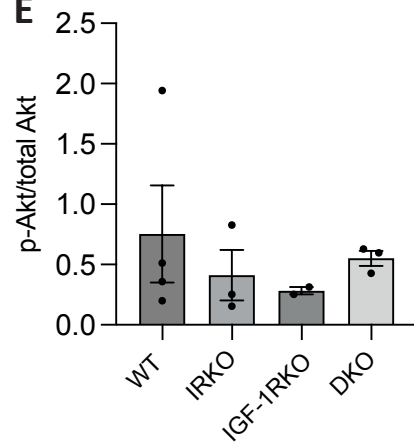

Supplement: Multimedia component 1 [file mmc1.pdf]
